# Thymic involution caused by repeated cocaine administration includes apoptotic cell loss followed by ectopic adipogenesis

**DOI:** 10.1371/journal.pone.0277032

**Published:** 2022-11-28

**Authors:** Kana Unuma, Homare Kaga, Takeshi Funakoshi, Moeka Nomura, Toshihiko Aki, Koichi Uemura

**Affiliations:** Department of Forensic Medicine, Graduate School of Medical and Dental Sciences, Tokyo Medical and Dental University, Tokyo, Japan; Tokyo University of Agriculture, JAPAN

## Abstract

Cocaine abuse has a negative impact on the immune system. To investigate the adverse effects of binge cocaine administration on lymphoid organs such as thymus and spleen, we examined the effects of repeated intravenous (i.v.) administration of cocaine on rats. Sprague Dawley rats (male, 8 weeks old) received 20 mg/kg body weight of cocaine hydrochloride per day for 7 or 14 days. In addition to a significant loss in the weight of the spleen, consistent with our previous intraperitoneal (i.p.) injection model of binge cocaine abuse (50 mg/kg cocaine for 7 days), we also found a significant loss of weight as well as apparent shrinkage of the thymus in the cocaine group. Transcriptome analysis of the thymus revealed increased expressions of genes involved in apoptosis, such as Ifi27 and Traf2, as well as decreased expressions of several genes related to lipid metabolism, such as Cd36, Adipoq, Scd1, and Fabp4, in the thymus of the cocaine group (7 days), suggesting an apoptotic loss of thymic cells as well as alterations in lipid metabolism. Paradoxically, cocaine activates PPARγ, a key transcriptional factor activating lipid metabolism, although ectopic adipogenesis was scarcely observed in the thymus. Further analysis of rats administered 20 mg/kg cocaine for 14 days revealed ectopic adipogenesis, which was accompanied with the activation of PPARγ as well as increased expression of Adipoq and Fabp4, in the thymus. Taken together, these results indicate that repeated cocaine administration induces thymic involution, which is initiated by the loss of thymic cells through apoptosis and subsequent ectopic adipocyte development.

## Introduction

Cocaine abuse is a worldwide problem for human health [1]. Although cardiovascular toxicity, as well as psychological effects resulting from alterations in the central nervous system, are the main toxicological features associated with cocaine abuse [2], cocaine also has harmful effects on the immune system [3]. For example, it has been observed that cocaine decreases the function of lymphocytes, including both T- and B-lymphocytes as well as natural killer cells, in mice [4]. Furthermore, cocaine also affects the immune system negatively, at least in part by altering the functional status of lymphoid organs including the thymus and spleen. For example, it has been shown both in vivo and in vitro that cocaine negatively regulates the proliferation of splenic T-lymphocytes as well as thymocytes [5, 6]. Recently, we showed that the intraperitoneal administration of 50 mg/kg cocaine once a day for a week results in sustained splenic contraction, although we did not examine the possibility that the thymus was also affected by this treatment [7].

Thymus is a lymphopoietic organ and plays a central role in the maturation of lymphocytes, which is necessary for adaptive immune reactions [8]. Due to its high sensitivity to stress, thymus is known to undergo shrinkage/involution in response to a wide variety of stimuli [9]. This acute stress-induced thymic involution is observed to occur in response to such factors as infection or chemotherapy with anti-tumor reagents. In general, acute stress-induced involution of the thymus is a result of the loss of thymic cells through apoptosis, and the subsequent reduction of thymic organ mass [9, 10]. The reduction of thymic mass can be reversed through proliferation of the remaining cells when the stimulus is eliminated, suggesting that stress-induced thymic involution is a reversible process [9, 11]. There is another type of thymic involution, called age-related thymic involution, that occurs during the development of almost all animals including humans [12, 13]. In contrast to stress-induced involution, thymic involution during aging is accompanied by ectopic adipogenesis, which fills the spaces lost during the involution [14].

Lipid metabolism is crucially involved in the determination of cellular activities, including those of immune cells [15]. Free fatty acids (FAs) are incorporated into cells through the membrane transporter complex consisting of molecules such as CD36 and FABP (fatty acid binding protein) [15]. Incorporated FAs are stored as triglycerides (TG) for future use as precursors of biomaterials including cholesterol and phospholipids or fuel to provide energy through oxidation (fatty acid oxidation, FAO) [16]. FAs are also synthesized intracellularly from acetyl-CoA and NADPH [16]. The balance between the storage and utilization of FAs is important for the fate of immune cells [15]. For example, the utilization of stored FA as fuel, referred to as lipolysis, through FAO in mitochondria is important for the survival of cells when glucose supplies are insufficient and mitochondrial respiration is decreased. In contrast, the storage of FA as TG in lipid droplets, referred to as lipogenesis, occurs in almost all types of cells, but most extensively in adipocytes [16]. Although alterations in lipid metabolism are implicated in the differentiation of T-lymphocytes as well as age-related thymic involution, little is known about the status of lipid metabolism in stress-induced acute thymic involution [15].

In this study, we examined the effects of repeated i.v. administration of cocaine in rats and found severe shrinkage of the thymus. This thymic involution is accompanied not only by apoptosis, but also by subsequent adipogenesis, suggesting that cocaine induces thymic involution with mixed features of both stress-induced and age-related involutions.

## Experimental procedures

All methods were carried out in accordance with relevant guidelines and regulations.

### Animals and the administration of cocaine

All animal experiments were approved by the Institutional Animal Care and Use Committee of Tokyo Medical and Dental University and performed in accordance with Animal Research: Reporting of In Vivo Experiments (ARRIVE) guidelines and regulations. In brief, rats were housed under a controlled temperature (25°C) and light/dark cycle (12h/12h) and given free access to water and food. Treatment of animals with cocaine was conducted as described previously [17]. In brief, rats (male, Sprague-Dawley, 8-week-old) were divided into two groups (control and cocaine groups, n = 4 in each group) and administered 20 mg/kg/day cocaine hydrochloride (cocaine group; Shionogi & Co., Ltd., Osaka, Japan, dissolved in saline) or the same volume of saline (control group) intravenously (i.v.) via the tail vein for 7 or 14 days. One day after the last administration, the rats were sacrificed by the administration of an overdose of anesthetic (40 mg/kg sodium pentobarbital). Heart, spleen, liver, left and right kidneys, brain, left and right lungs, and thymus were excised from the sacrificed animals. The organs were weighed and stored at -20°C (for protein extraction) or -80°C (for RNA extraction). For tissue staining, pieces of organs were fixed in 4% paraformaldehyde (PFA).

### Staining of tissues

After fixation with PFA, tissues were embedded in paraffin, sliced into 2.5 μm thick specimens, subjected to hematoxylin-eosin (H&E) staining, and observed under a light microscope (AX-80, Olympus, Tokyo, Japan). Formalin fixed frozen sections were stained with the Oil Red-O.

### DNA microarray analysis

Total RNA extracted in Trizol reagent (Thermo) was further purified using an RNeasy kit (Qiagen, USA). The resultant purified total RNA was subjected to DNA microarray analysis using a Clariom^TM^S array (Thermo). The datasets were analyzed by Transcriptome Analysis Console (TAC) software (Thermo).

### Immunoblotting

Tissues were lysed in STE buffer [320 mM sucrose, 10 mM Tris-HCl, 5 mM EDTA, 50 mM NaF, 2 mM Na_3_VO_4_, and protease inhibitor cocktail (Roche Diagnostics, Mannheim, Germany)], and equal amounts of the lysates were subjected to SDS-PAGE. Immunoblotting was performed with the antibodies listed in S1 Table. Peroxidase-conjugated anti-mouse or rabbit IgG antibodies (Promega Corporation, USA) and enhanced chemiluminescence reagents (Thermo Fisher Scientific, USA) were used to visualize antigens. Images of the blots were captured by LuminoGraph III (ATTO, Tokyo, Japan). CS analyzer 4 image analyzing software (ATTO, Tokyo, Japan) was used to quantify the densities of the bands. Uncropped gel images are found in S1 Raw images.

### Plasma protein preparation for immunoblot analysis

Blood was collected through cardiac puncture followed by removal of the albumin using a Pierce Albumin Depletion Kit (Thermo).

### Quantitative reverse transcriptase-mediated real time PCR

Total RNA was extracted from the tissues using Trizol reagent. Reverse transcription was performed using SuperScript II and oligo(dT)_15_. StepOne Plus real time PCR was used to quantify the mRNA levels of the samples. SYBRgreen was used as a fluorescence dye and a comparable Ct method was adopted to measure the relative mRNA levels. The primers used are listed in S1 Table.

### Electron microscopy

Transmission electron microscopy (TEM) analysis was performed as described previously. In brief, specimens of thymus were washed with 0.1 M phosphate buffer (PB), fixed sequentially with 4.5% paraformaldehyde and 2.5% glutaraldehyde in PB, incubated in 1% osmium tetroxide, dehydrated in a series of graded ethanol solutions, and embedded in Epon epoxy resin. Ultrathin sections of the embedded specimens were stained with uranyl acetate and lead citrate, and observed under a transmission electron microscope (H7100, Hitachi, Japan) equipped with an AMT Advantage-HS CCD camera (AMT, Woburn, USA).

### Blood analysis

Blood samples were obtained from the rats via the caudal veins. Serum total cholesterol (T-Cho), triglyceride, and glucose levels were analyzed using standard methods of the Oriental Yeast Co., Ltd. (Tokyo, Japan).

### Immunohistochemistry

Immunohistochemical analysis of thymus was performed as follows. Tissue sections were deparaffinized and antigen retrieved by heating. To terminate the endogenous peroxidase activity, 3% hydrogen peroxide was applied to the sections. After blocking with 0.5% bovine serum albumin serum in PBS, the sections were incubated with antibodies overnight at 4°C. Antigens were detected with Histofine Simple Stain MAX-PO (MULTI) kit (Nichirei Biosciences Inc., Tokyo, Japan), and visualized by 3,3’-diaminobenzidine (Nichirei Biosciences Inc.).

### Cellular triglyceride levels

Relative cellular triglyceride levels were determined using Triglyceride-Glo Assay kit (Promega, Madison, WI).

### Statistical analysis

Student’s *t*-test was used to assess statistical significance throughout this study. Statistical differences were considered significant at *p* < 0.05.

## Results

### Repeated cocaine administration results in significant weight loss of thymus

**Fig 1:**
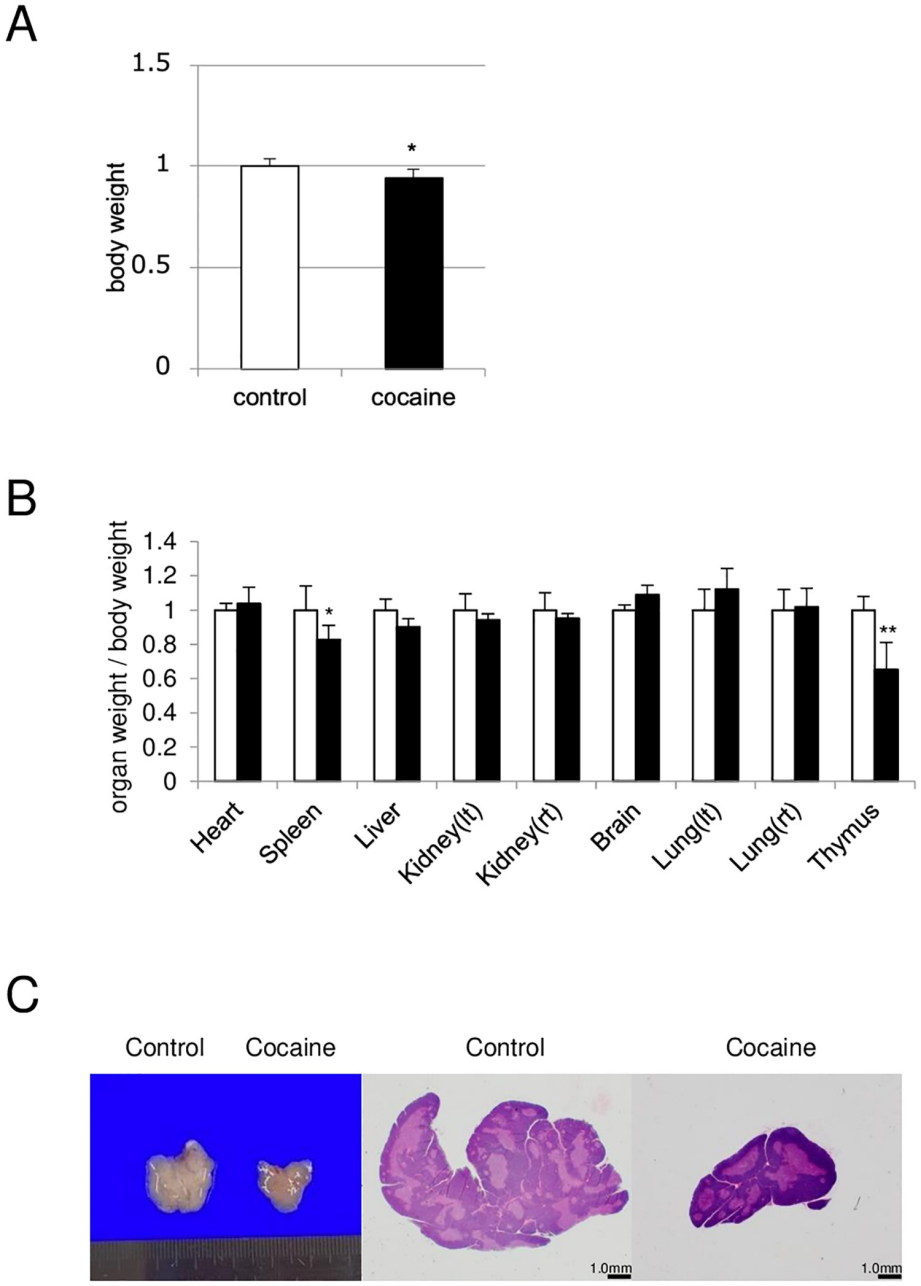
Reduction of thymic weight and size in rats administered cocaine. Rats were treated with or without cocaine (20 mg/kg/day, 7 days). (A) Loss of body weight in rats administered cocaine. (B) Changes in relative weights of major organs in rats administered cocaine. Lt, left. Rt, Right. For the graphs in (A) and (B), mean relative weights of the control group were set to 1. (C) Reduction of the thymic size in rats administered cocaine. Light microscopic and H&E staining images of thymus in rats administered cocaine are shown. Graphs show mean and S.E. **P<0.01, *P<0.05 versus control.

As described in “Introduction”, we have shown shrinkage of the spleen in our previous i.p. model of cocaine abuse [7]. To further examine the effects of repeated cocaine administration, we adopted intravenous injections (i.v.) of cocaine in this study and examined changes in the weights of major organs: heart, spleen, liver, left and right kidney, brain, left and right lungs, and thymus. As observed in our previous i.p. model, significant reductions in body weight and spleen weight were also observed in the present study (Fig 1). In addition, we also observed a significant decrease in the relative weight of the thymus, a lymphoid organ not examined in our previous study [7] (Fig 1). Not only the weight but also the apparent size of the thymus was reduced, suggesting involution/atrophy of this organ (Fig 1). Since it is well-known that the thymus is highly vulnerable to stresses such as infection and chemotherapy [9], these results indicate that repeated cocaine administration also causes stress in rats and eventual thymic involution.

### Transcriptome analysis of cocaine-induced alterations in gene expression in thymus

**Fig 2:**
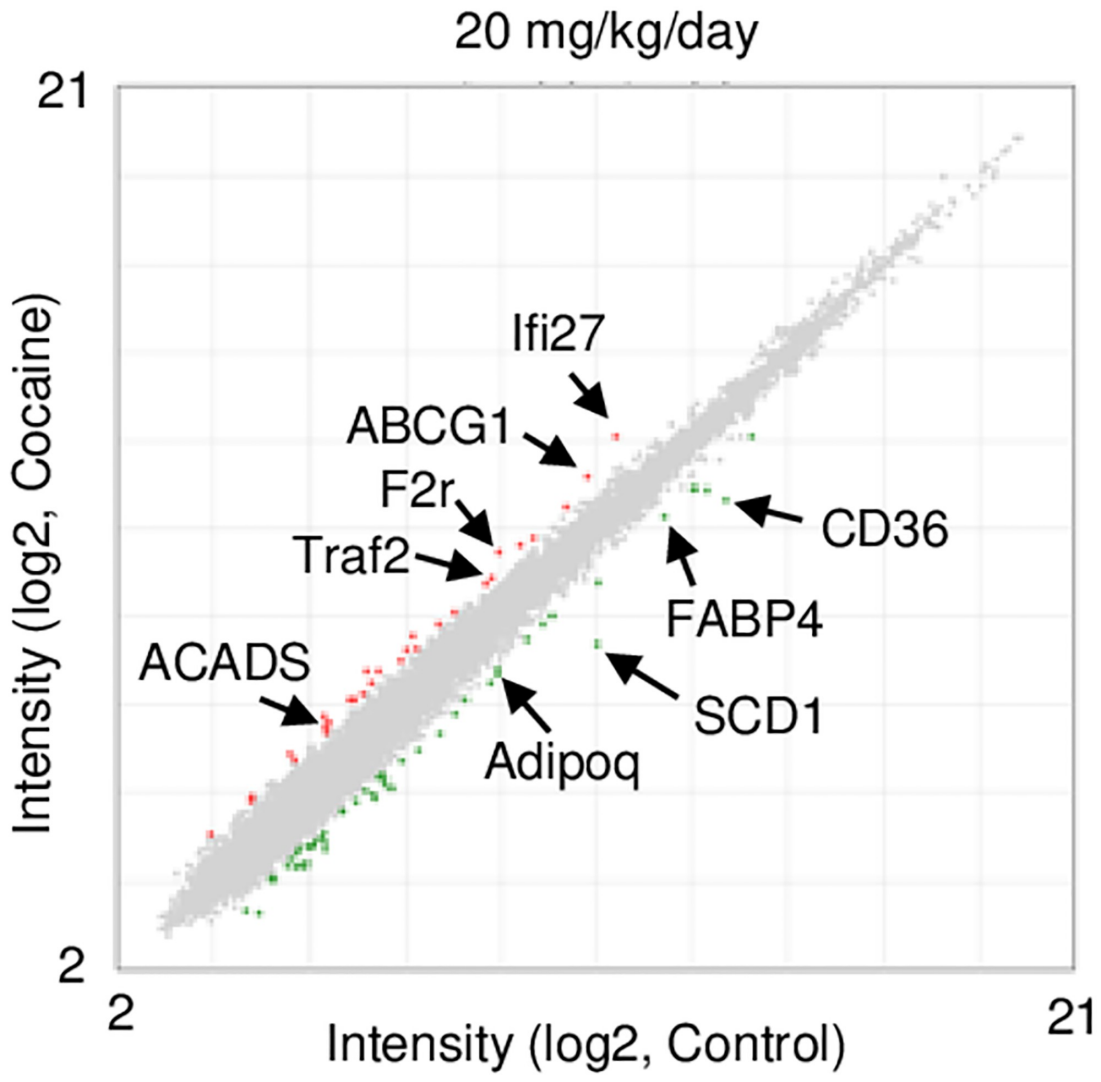
Transcriptome analysis of the thymus in rats administered cocaine. Rats were treated with or without cocaine (20 mg/kg/day, 7 days). Total RNA was extracted from the thymus and subjected to DNA microarray analysis. The scatter plot shows transcripts upregulated (red circles) and downregulated (green circles) by more than 2-fold by cocaine treatment.

To gain insight into the mechanism underlying thymic involution in cocaine-administered rats, transcriptome analysis using DNA microarray was performed (Fig 2). Among the genes upregulated more than 2-fold following the administration of 20 mg/kg/day cocaine for 7 days (Fig 2), Ifi27/Isg12a (interferon alpha inducible protein 27/interferon-stimulated genes 12a) and Traf2 (TNF receptor-associated factor 2) have been shown to be involved in apoptosis [18, 19]. Increased expressions of several genes involved in lipid metabolism were also found (Fig 2); Acads (acyl-CoA dehydrogenase short chain) is involved in the β-oxidation of fatty acids in mitochondria [20] while Abcg1 (ATP-binding cassette subfamily G member 1) mediates cholesterol efflux from cells [21]. On the other hand, decreased expressions of genes involved in FA transport into cells, Cd36 and Fabp4 (fatty acid binding protein 4) [15], were observed in cocaine-administered rat thymus. Scd1 (stearoyl-CoA desaturase 1), which is involved in FA synthesis [22], was also decreased in the cocaine group. Furthermore, decreased expression of Adipoq (adiponectin), an adipokine mainly secreted from adipocytes [23, 24], was also observed. Collectively, these results suggest 1) apoptosis in thymocytes, as well as 2) alterations in FA metabolism in cocaine-administered rat thymus. Since both apoptosis and FA metabolism are implicated in thymic involution, we examined them further.

### Repeated cocaine administration induces apoptosis but not autophagy and cell cycle arrest in the thymus

**Fig 3:**
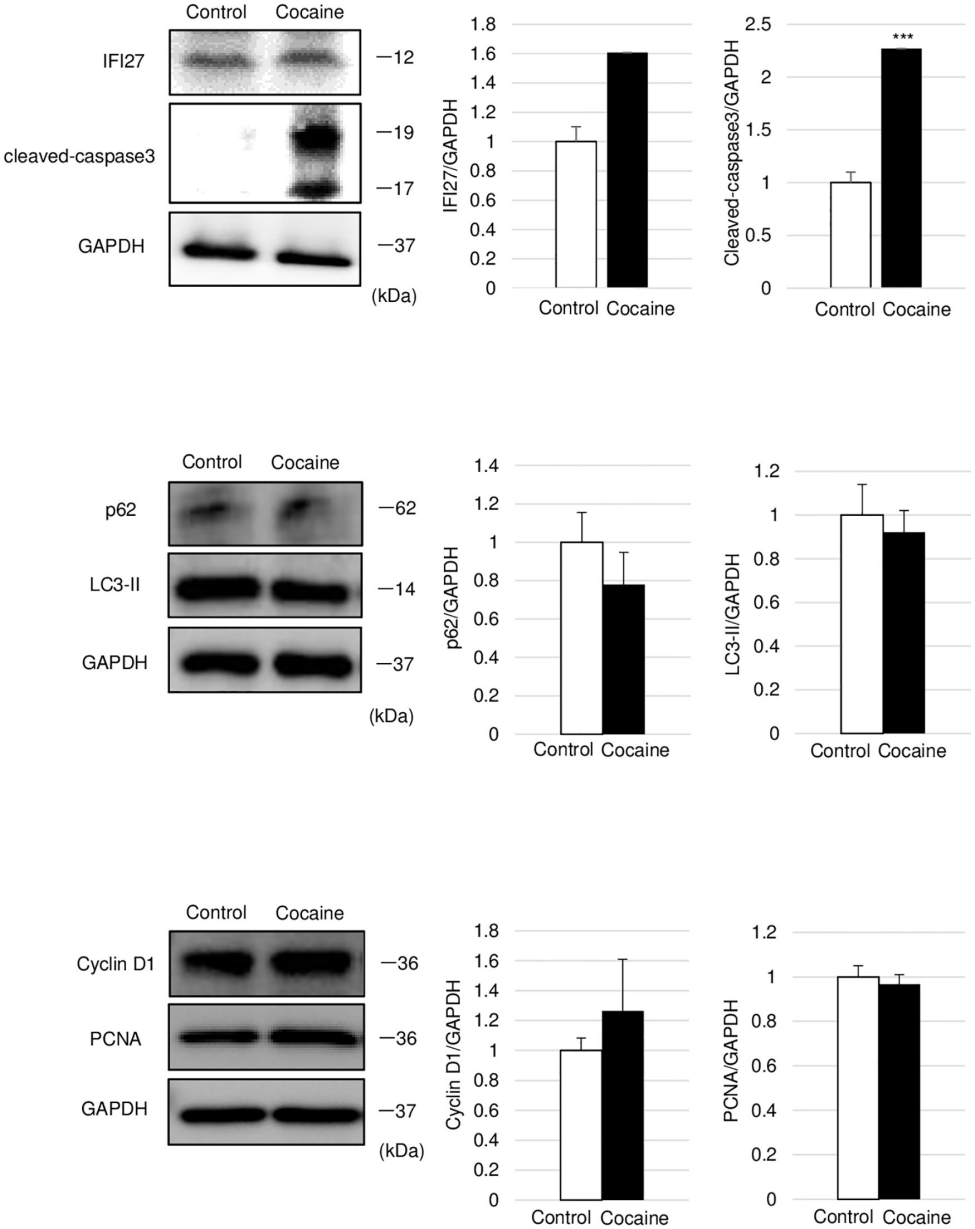
Apoptosis in thymus from cocaine-administered rats. Rats were treated with or without cocaine (20 mg/kg/day, 7 days). Proteins were extracted from the thymus and subjected to immunoblot analysis. Markers of apoptosis (IFI27 and cleaved-caspase 3), cell cycle (cyclin D1 and PCNA), and autophagy (LC3 and p62) are shown. GAPDH levels served as an internal control. Graphs show mean and S.E. ***P<0.001 versus control.

Given the indication from the results of DNA microarray analysis of thymocyte apoptosis following repeated cocaine administration (Fig 2), we examined cocaine-administered rat thymus for signs of apoptosis. Immunoblot analysis showed significant increases in the levels of not only Ifi27, but also cleaved caspase-3 (p17 and p19), the active forms of this enzyme, in the cocaine group as compared to the control group (Fig 3). In contrast, we observed no differences between the levels of p62 and LC3-II, autophagy markers, denying the involvement of autophagy (Fig 3). Despite the previous reports demonstrating decreased proliferation of thymic cells in cocaine-administered mice [6], there were no significant differences in the levels of cell the proliferation markers PCNA and cyclin D1 between the cocaine and control groups (Fig 3). Therefore, apoptosis should contribute to the atrophy of thymus in cocaine-administered rats while autophagy and the cell cycle are scarcely involved.

### Repeated cocaine administration induces a slight development of adipose tissue and an alteration of mitochondrial dynamics towards fission in the thymus

**Fig 4:**
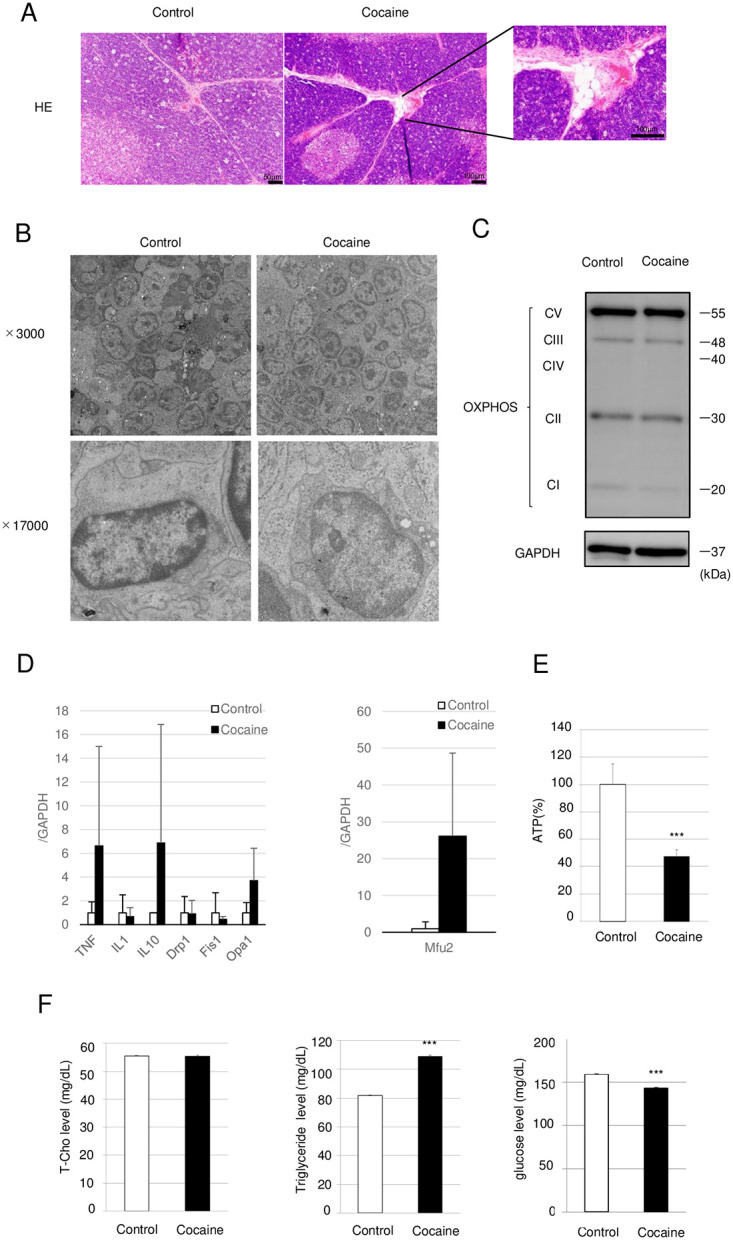
Ectopic development of a small number of adipocytes, as well as mitochondrial morphology and dynamics, in thymus from cocaine-administered rats. Rats were treated with or without cocaine (20 mg/kg/day, 7 days). (A) H&E staining images of thymus are shown. Rats were treated with or without cocaine (20 mg/kg/day, 7 days). (B) Electron microscopic images of thymus from rats treated with or without cocaine. (C) Immunoblot analysis of OXPHOS proteins. (D) Quantitative real time PCR analysis of genes involved in mitochondrial dynamics. GAPDH levels served as an internal control. (E) Relative ATP levels in the thymus from rats treated with or without cocaine. (F) Serum concentrations of total cholesterol (T-Cho), triglyceride, and glucose. Graph shows mean and S.E. ***P<0.001 versus control.

We observed changes in lipid metabolism (Fig 2), and age-related thymic involution involves the replacement of thymic cells with adipocytes [14]. Therefore, we examined whether ectopic adipogenesis could be observed in the thymus of rats administered 20 mg/kg cocaine for 7 days. H&E staining of the thymus showed only a trace-level deposition of adipocytes in the trabeculae as well as subcapsular sinuses of thymus from rats administered cocaine (Fig 4A). Thus, the cocaine-induced atrophy of the thymus does not have the features of age-related thymic involution, at least after repeated cocaine administration for 7 days. We next examined mitochondria, as mitochondrial toxicity is one of the most important mechanisms of cocaine cytotoxicity [25], and there is an intimate connection between mitochondria and apoptosis, as well as FA metabolism. Electron microscopic analysis indicated a normal morphology of the mitochondria in the cocaine group, suggesting mitochondrial homeostasis might be maintained even after repeated cocaine administration (20 mg/kg, 7 days) (Fig 4B). Immunoblot analysis of OXPHOS proteins also confirmed that the levels of electron transport chain (ETC) proteins were unchanged between the control and cocaine groups (Fig 4C). There are close relationships between mitochondrial function and dynamics, and previous reports including ours have shown that cocaine alters mitochondrial dynamics in murine neuronal cells and rat hearts [17, 26, 27]. We therefore examined whether repeated cocaine administration also affects mitochondrial dynamics in the thymus. As shown in Fig 4D, increased expressions of the genes for mitofusin2 and OPA1, which are involved in the fusion of mitochondria [28, 29],was observed in the cocaine group as compared to the control group. In addition, a trend toward the decreased expressions of Drp1 and Fis1 was also observed, suggesting a tendency toward mitochondrial fusion in the thymus of the cocaine group (Fig 4D). We also observed a significant increase in the expression of cytokines (TNFα and IL-10), suggesting the presence of an inflammatory response. Furthermore, there were significant decreases in relative ATP levels in the thymus from cocaine-treated rats compared to that of control rats, suggesting a decrease in mitochondrial function (Fig 4E). Thus, cocaine alters mitochondrial dynamics toward fusion, which might contribute to the maintenance of mitochondrial homeostasis in rat thymus during cocaine administration. We finally examined serum levels of total cholesterol, triglyceride, and glucose, and found that triglyceride and glucose levels were increased and decreased, respectively, in rats treated with cocaine (Fig 4F). Since glucose is needed to generate cellular fatty acids as well as triglycerides, this alteration in the blood might be related to the change towards adipogenesis in the thymus.

### Repeated cocaine administration activates PPARγ in the thymus

**Fig 5:**
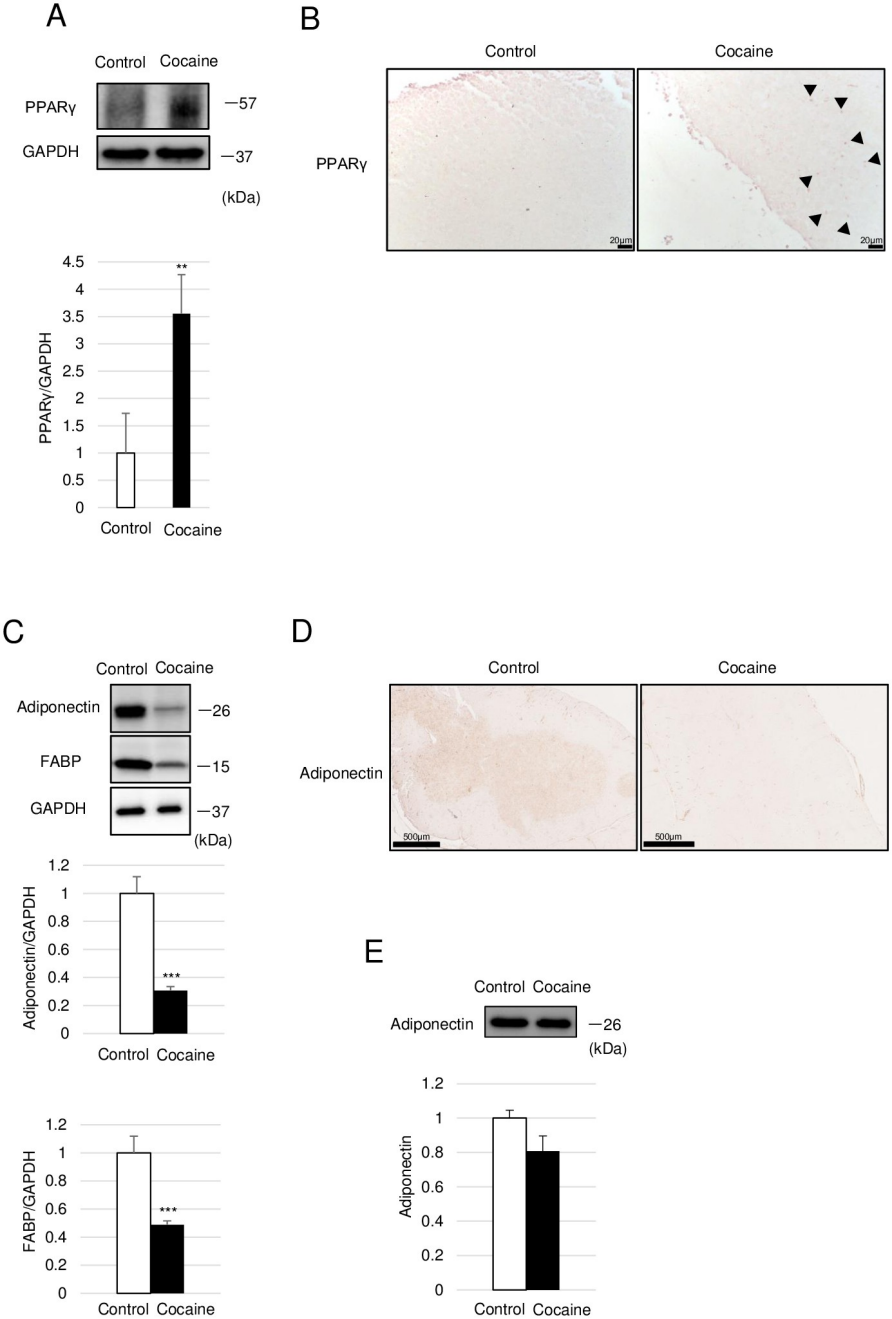
Increased levels of PPARγ and decreased levels of adiponectin in thymus from rats administered cocaine for 7 days. Rats were treated with or without cocaine (20 mg/kg/day, 7 days). (A) Immunoblot analysis of PPARγ in the thymus. GAPDH levels served as an internal control. (B) Immunohistochemical analysis of PPARγ in the thymus. Arrowheads indicate nuclear localization of PPARγ. (C) Immunoblot analysis of adiponectin and FABP4 in the thymus. (D) Immunohistochemical analysis of adiponectin in the thymus. (E) Immunoblot analysis of adiponectin in the serum. GAPDH levels served as an internal control. Graphs show mean and S.E. **P<0. 01, ***P<0.001 versus control.

FA metabolism is regulated mainly through the peroxisome proliferator-activated receptor (PPAR) family of transcription factors. Among them, PPARγ plays a central role in FA uptake and subsequent adipogenesis [30]. Therefore, we examined PPARγ in the thymus. As shown in Fig 5A, increased protein levels of PPARγ were observed in the thymus of the cocaine group. Immunohistochemistry also showed the nuclear translocation of PPARγ in several thymic cells in the cocaine group, confirming the activation of PPARγ by cocaine in the thymus (Fig 5B). We next examined the decrease in adiponectin as well as FA intake genes indicated in the results of the DNA microarray analysis (Fig 2). We performed immunoblotting for adiponectin and FABP4, as an example of a FA intake-related protein, as well as an immunohistochemical analysis of adiponectin. As shown in Fig 5C, the protein levels of adiponectin and FABP4 were significantly decreased in the cocaine group as compared to the control group. Immunohistochemical analysis also showed an apparent decrease in adiponectin staining within the medulla of the thymus in the cocaine administered rats as compared to controls (Fig 5D). Since adiponectin circulates throughout the body, we examined circulating levels of adiponectin, and found no difference in the levels of adiponectin in blood samples from the cocaine and control groups (Fig 5E). Thus, adiponectin seems to be decreased specifically in the microenvironment of the thymic medulla in cocaine-administered rats. Taken together, PPARγ seems to be activated in the thymus of rats administered cocaine for 7 days, in spite of the decreases in its target genes, such as Fabp4 (Figs 2 and 5).

### Repeated cocaine administration for 14 days induces ectopic development of adipose tissue in the thymus

**Fig 6:**
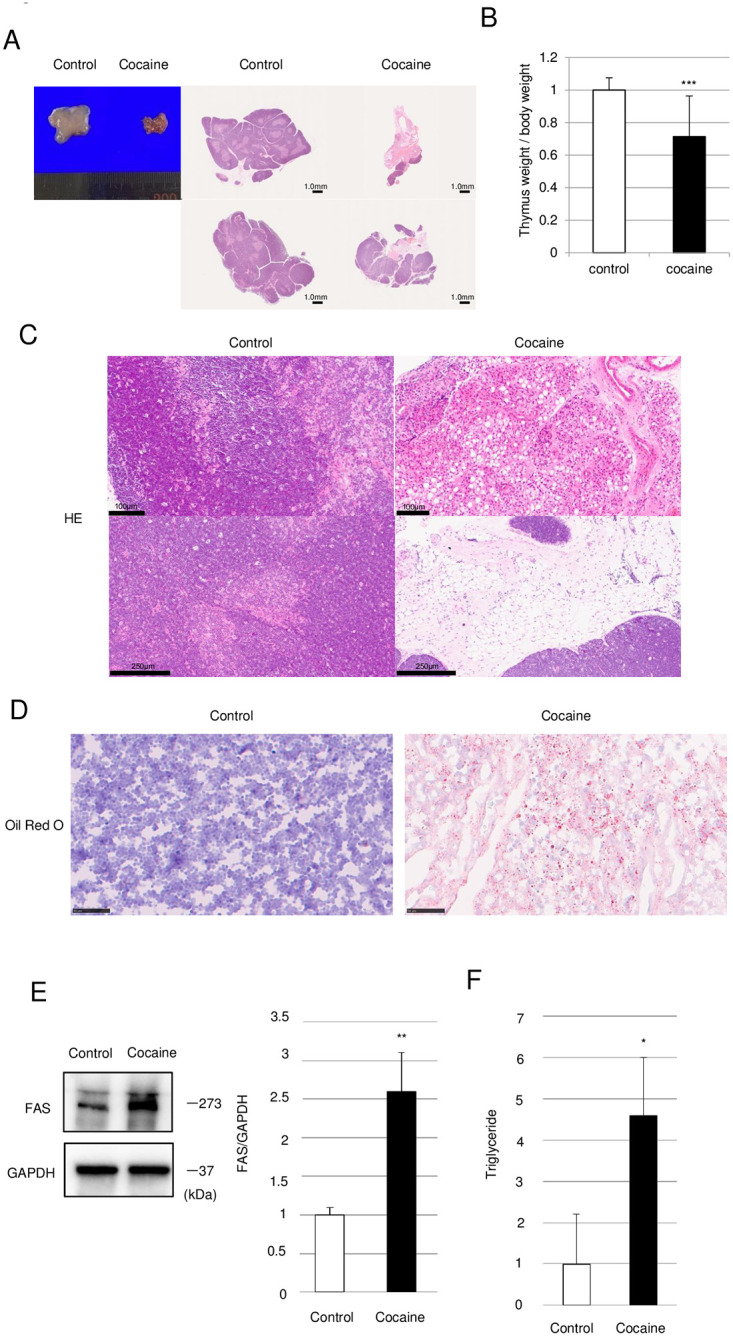
Reduction of thymic weight and size as well as ectopic development of adipocytes in thymus from rats administered cocaine for 14 days. Rats were treated with or without cocaine (20 mg/kg/day, 14 days). (A) Persistence of the reduction in thymic size in rats administered cocaine. Light microscopic and H&E staining images of thymus from rats administered cocaine for 14 days are shown. (B) Reduction in relative weight of thymus from rats administered cocaine for 14 days. (C) Ectopic development of adipocytes in thymus of rats administered cocaine for 14 days. Massive development of adipocytes was observed at the periphery of and within the cortex of the thymus. (D) Oil Red O staining images of thymus from rats administered cocaine for 14 day. (E) Immunoblot analysis of fatty acid synthase (FAS) in the thymus. GAPDH levels served as an internal control. (F) Relative levels of triglycerides in the thymus. Graph shows mean and S.E. *P<0.05, **P<0.01, ***P<0.001 versus control.

Age-related thymic involution involves the replacement of thymic cells with adipocytes. As a natural consequence of its crucial role in the development of adipose tissue [31], PPARγ also play a pivotal role in the age-related involution of the thymus [32, 33]. However, we did not observe any appreciable development of adipose tissue in the thymus of rats administered cocaine for 7 days (Fig 4), despite the activation of PPARγ (Fig 5). Therefore, we examined the thymus of rats administered cocaine for 14 days. As shown in Fig 6A and 6B, shrinkage as well as the loss of weight of the thymus were observed, suggesting that thymic involution continued during days 8–14 cocaine administration. Furthermore, along with the loss of thymic cells, such as epithelial cells, large clusters of adipocytes were observed sporadically in the thymus of the cocaine group (Fig 6C). There also seemed to be a replacement of lymphoid cells with such clusters of adipocytes (Fig 6C and 6D). In accordance with the observed histological changes, we also observed increases in the levels of fatty acid synthase (FAS) and cellular triglycerides in the thymus from rats administered cocaine for 14 days (Fig 6E and 6F). These results suggest that the activation of PPARγ observed in the thymus from rats administered cocaine for 7 days (Fig 5) should lead to later ectopic adipogenesis, characteristic of age-related thymic involution.

### Repeated cocaine administration for 14 days increases PPARγ-target gene expressions in the thymus

**Fig 7:**
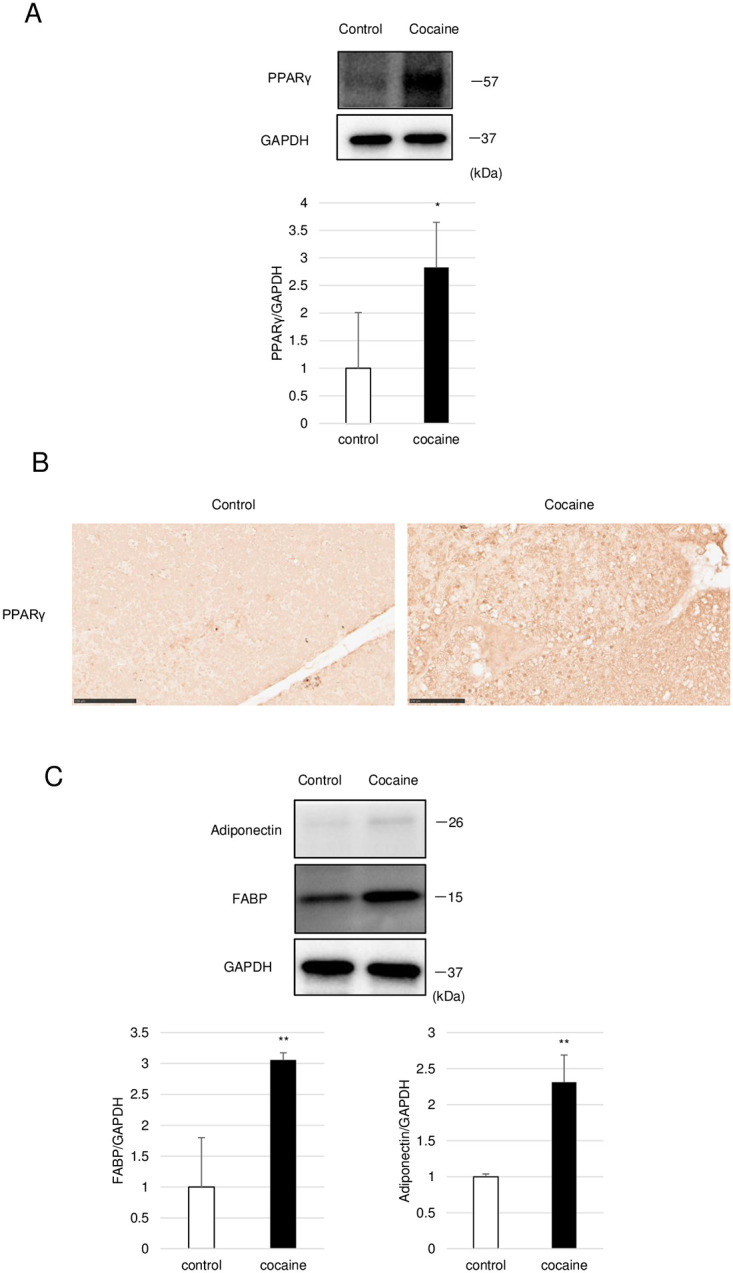
Increased levels of PPARγ as well as adiponectin in thymus from rats administered cocaine for 14 days. Rats were treated with or without cocaine (20 mg/kg/day, 14 days). (A) Immunoblot analysis of PPARγ in the thymus. GAPDH levels served as an internal control. (B) Immunohistochemical analysis of PPARγ in the thymus. Arrowheads indicate nuclear localization of PPARγ. (C) Immunoblot analysis of adiponectin and FABP in the thymus. GAPDH levels served as an internal control. Graphs show mean and S.E. *P<0. 05, **P<0. 01 versus control.

As observed in the thymus of rats administered cocaine for 7 days, we observed increased expression as well as nuclear translocation of PPARγ in the thymus of rats with 14 days cocaine treatment (Fig 7A and 7B). Furthermore, the activation was accompanied by increased protein levels of FABP4 and adiponectin (Fig 7C). Thus, after 14 days administration in rats, the activation of PPARγ seems to be associated with the increase of PPARγ-target genes as well as apparent adipogenesis in the thymus.

## Discussion

In this study, we demonstrate that repeated cocaine administration induces shrinkage of the thymus. Although involution of the thymus has been repeatedly reported in animal models of cocaine abuse [6, 34], this is the first study, to our best of knowledge, to demonstrate that acute shrinkage is followed by ectopic development of adipocytes in the thymus during prolonged cocaine treatment in rats.

During thymic involution caused by cocaine, the number of cells in the thymus decreases as a prerequisite for the loss of thymic volume. To explain this decrease in cell number, two distinct, but not mutually exclusive, mechanisms have been suggested: a loss of thymic cells through apoptosis and the suppression of cell proliferation [10, 35]. Our results shown in Fig 3 demonstrate that apoptosis rather than cell cycle arrest contributes to the loss of cells and subsequent involution of the thymus. Thus, it seems that repeated daily cocaine administration causes acute stress-induced thymic atrophy within 7 days after the beginning of exposure. Apoptotic loss of thymic cells is responsible, at least in part, for the atrophy of this organ observed at this stage.

In the thymus of rats administered cocaine for 7 days, thymic atrophy is accompanied by a slight, sporadic occurrence of adipocytes in the trabeculae (Fig 4). In contrast, this acute stress-induced atrophy was followed by obvious ectopic adipogenesis, which occurred mainly during days 8–14 after the initiation of cocaine treatment. Replacement of stromal cells with adipocytes is one of the characteristic features of age-related thymic atrophy, which occurs as a result of aging even in healthy individuals. However, the 8–10 week-old rats used in this study are still in the adolescent or young adult stage [36]. Thus, it may be safe to conclude that repeated daily cocaine administration accelerates age-related thymic atrophy in rats.

PPARγ has been shown to be a direct transcriptional activator of several genes for FA uptake including Cd36 and Fabp4 [37]. However, on day 7 after the start of cocaine treatment, we observed decreases in the expressions of Cd36 and Fabp4 in the thymus despite the histological as well as biochemical observation demonstrating the activation of PPARγ (Figs 2 and 5). Since the activation of PPARγ is consistent with, and should be required for, the following adipogenesis, the paradoxical decrease in the expressions of its target genes might indicate that the expressions of these genes were more profoundly decreased at an earlier treatment stage (1–7 days treatment). In other words, the expressions of these genes might have been in a recovery period when the thymus was examined at day 7 of cocaine treatment. In accordance with this assumption, we observed not only PPARγ activation but also increased expression of adiponectin and FABP in the thymus after administration of cocaine for 14 days (Fig 7). Alternatively, there might be two types of thymic cells, one that undergoes differentiation into adipocytes through the facilitation of FA intake while the other rather consumes and/or excretes intracellular FA. In any case, there appears to be complicated alterations in FA metabolism during thymic atrophy caused by repeated cocaine administration in rats.

In addition to the paradoxical decrease in the expressions of the genes for FA intake, we also observed a decrease in the expression of adiponectin in our DNA microarray analysis. Indeed, levels of the adiponectin protein were severely decreased in the thymus of cocaine-administered rats. Since the circulating levels of adiponectin were unchanged between the control and cocaine groups (Fig 5E), the downregulation of adiponectin to occur locally in the microenvironment of the thymus. It has been reported that lymphocytes [38] as well as certain other cells within thymus [39] express the AdipoQ gene. However, given the limited expression of AdipoQ in these cells as well as the fact that AdipoQ is highly abundant in circulation, severe and apparent decrease in AdipoQ as observed by immunoblot and histochemistry, respectively, may be attributable to a loss of this protein in the extracellular microenvironment of the thymus. Takemura, et al. reported that adiponectin is involved in the clearance of apoptotic cells from the thymus [24].Therefore, the loss of adiponectin might result from the occurrence of apoptosis in the thymus and subsequent clearance along with apoptotic cells by macrophages. Since the levels of adiponectin is the thymus were rather increased in rats administered cocaine for 14 days, decreased levels of adiponectin in the thymus should be transient and might be a result from transient occurrence of apoptosis in the thymus.

Age-related atrophy of the thymus is believed to play a significant role in immunosenescence, the phenomenon in which immune ability declines with age. Like aging, cocaine abuse has also been reported to have negative effects on the immune system. Nevertheless, a rational explanation for the negative effect of cocaine on the immune system has scarcely been reported. Our current results showing that cocaine accelerates at least one feature of age-related thymic involution, ectopic adipogenesis, may provide an important clue to elucidate the whole mechanism of the negative effects of cocaine on the immune system.

## Supporting information

S1 Raw images (PDF)

S1 Table (PDF)
