# Supplementary material for: Thymic involution caused by repeated cocaine administration includes apoptotic cell loss followed by ectopic adipogenesis
Source: PLoS One. 2022 Nov 28;17(11):e0277032. doi: 10.1371/journal.pone.0277032 (PMC9704633; doi:10.1371/journal.pone.0277032)
Supplement: S1 Raw images — (PDF) [file pone.0277032.s001.pdf]

The full length blots of Fig. 3

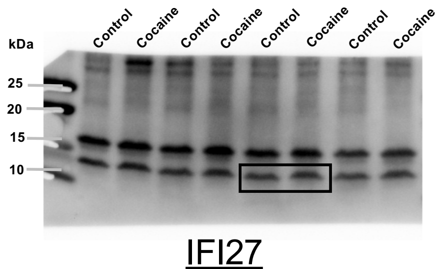

IFI27

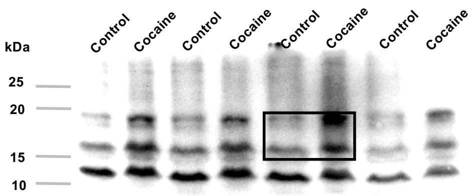

cleaved-caspase3

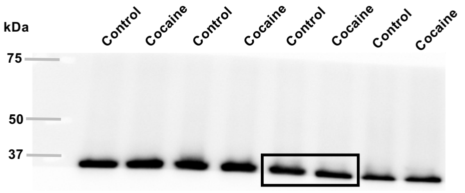

GAPDH

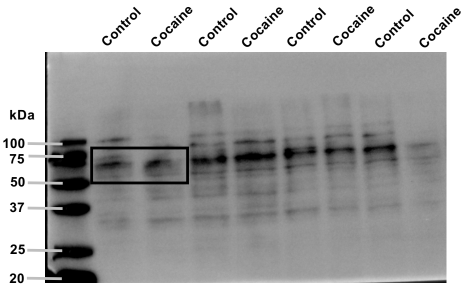

p62

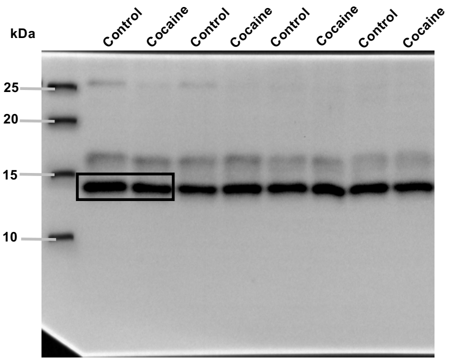

LC3-II

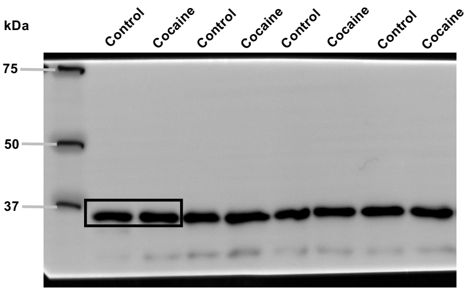

GAPDH

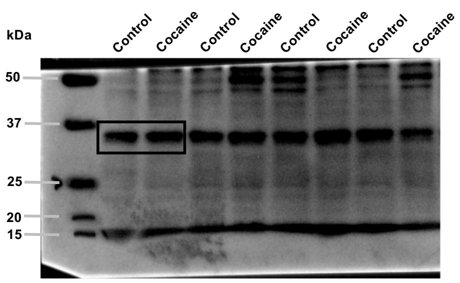

Cyclin D1

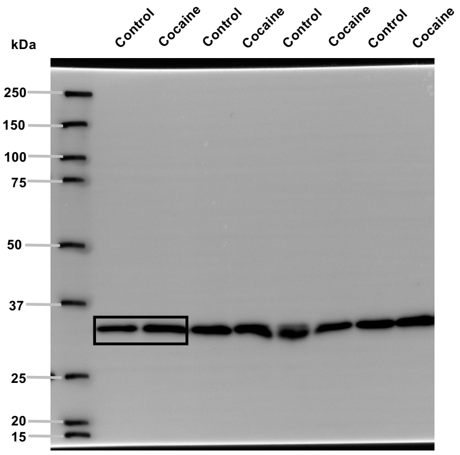

PCNA

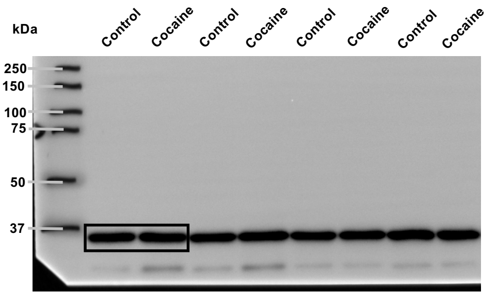

GAPDH

The full length blots of Fig. 4

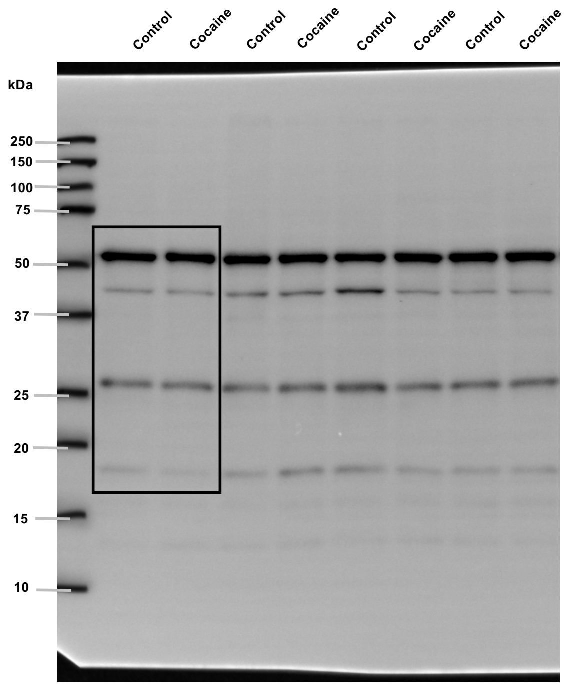

OXPHOS

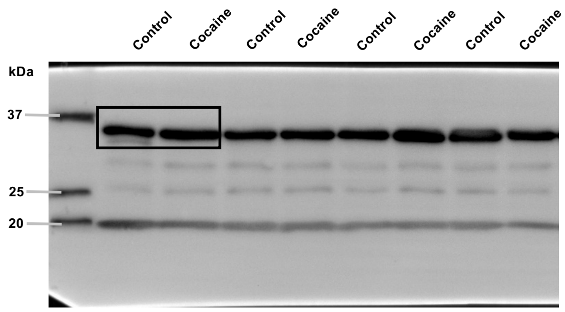

GAPDH

The full length blots of Fig. 5

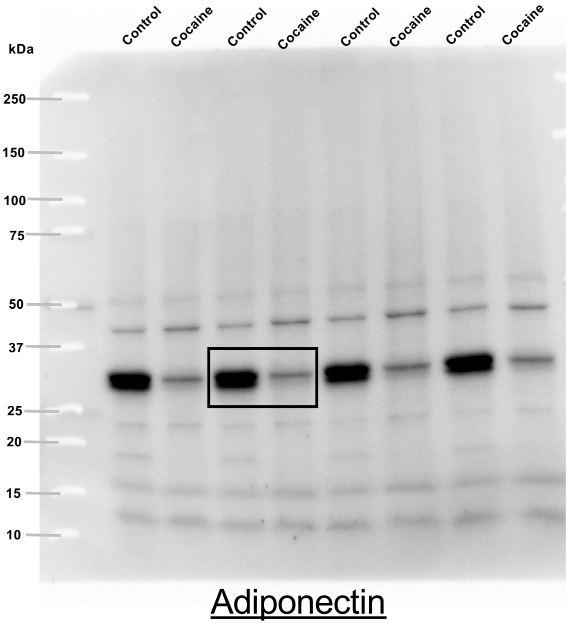

Adiponectin

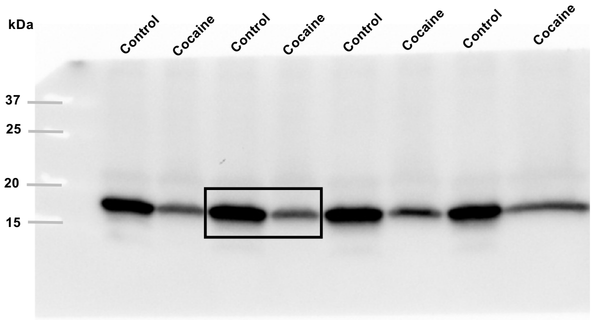

FABP

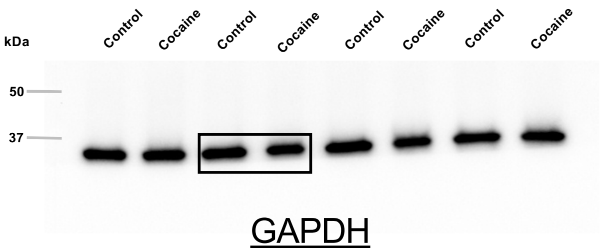

GAPDH

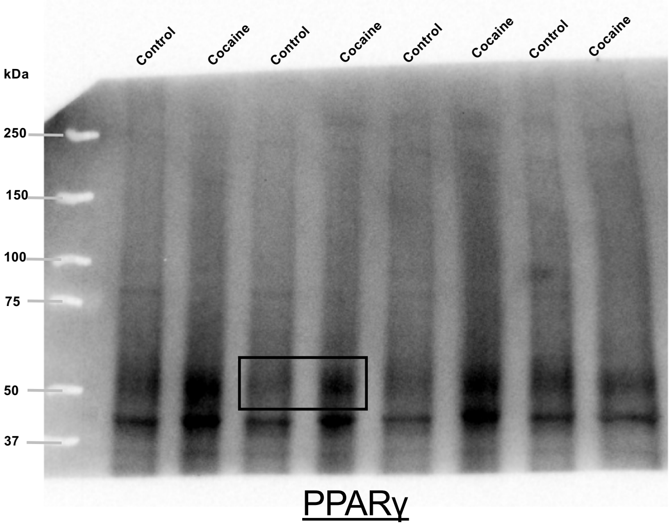

PPARγ

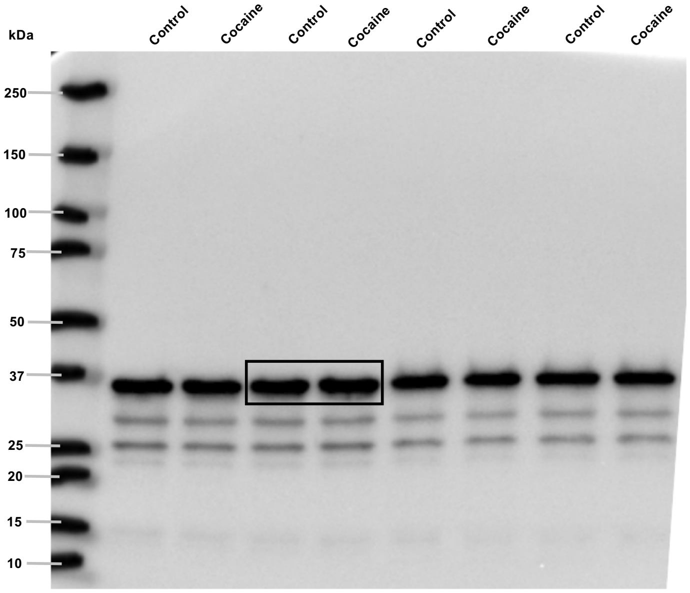

GAPDH

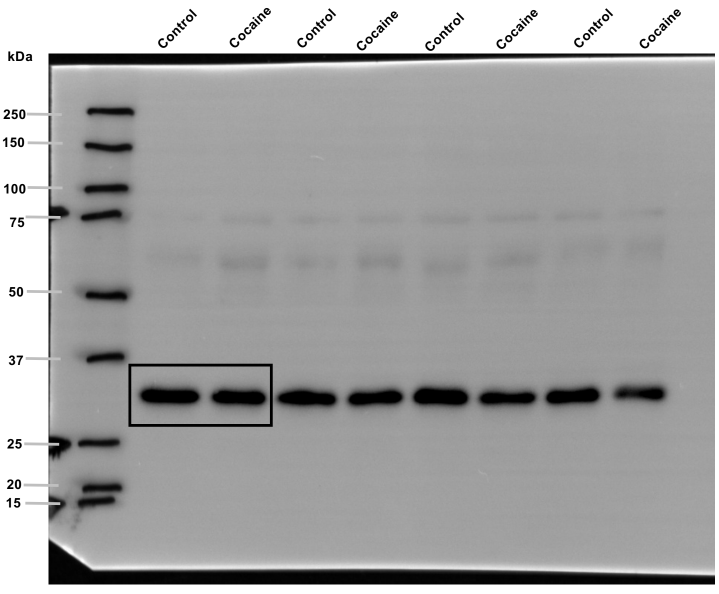

Adiponectin

The full length blots of Fig. 6

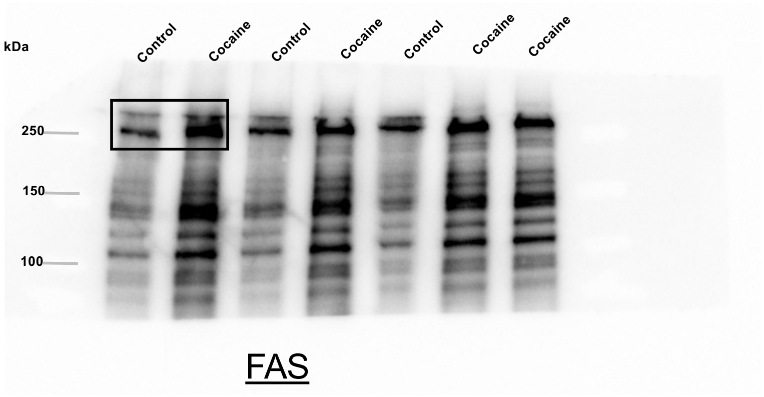

FAS

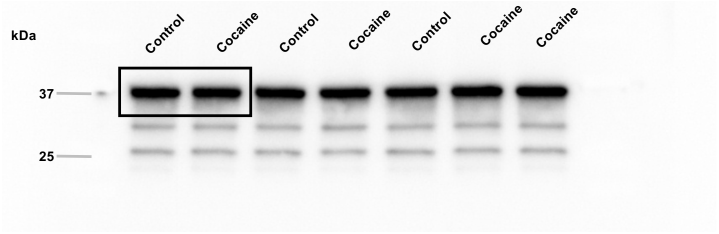

GAPDH

The full length blots of Fig. 7

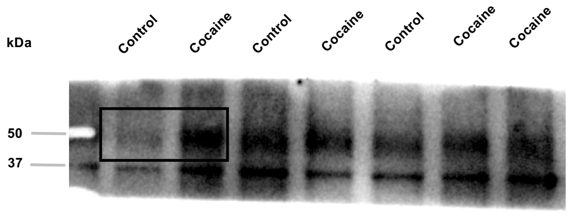

PPARγ

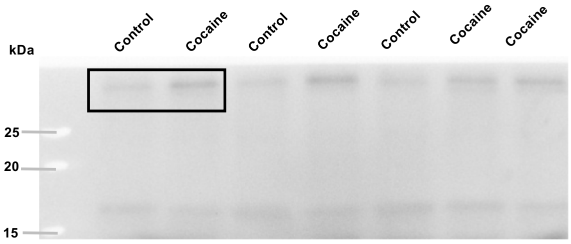

Adiponectin

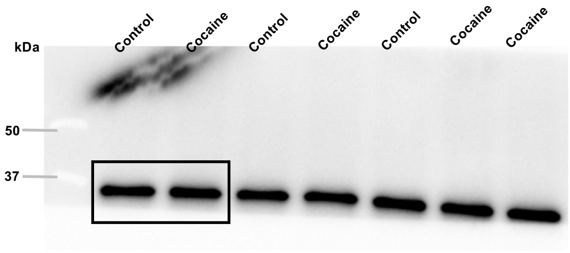

GAPDH
