# Supplementary material for: Thymic involution caused by repeated cocaine administration includes apoptotic cell loss followed by ectopic adipogenesis
Source: PLoS One. 2022 Nov 28;17(11):e0277032. doi: 10.1371/journal.pone.0277032 (PMC9704633; doi:10.1371/journal.pone.0277032)
Supplement: S1 Table — (PDF) [file pone.0277032.s002.pdf]

Primers used in this study

Gene	Forward	Reverse
TNF-α	ATGGCCTTGTAGACACCTTGG	ATGGCCTTGTAGACACCTTGG
IL-1β	ATGGCCTTGTAGACACCTTGG	ATGGCCTTGTAGACACCTTGG
IL-10	AATAAAAGCAAGGCAGTGGAGC	ATTCATGGCCTTGTAGACACCT
Drp1	TTCTTCCCAGAGGGACTGGT	GAAATTTACCCCATTCTTCTGCT
Fis1	GGGTTACATGGATGCCCAGA	AGGCACCAGGCGTATTCAAA
Mfn2	CACCCGGGATAATCTGGAGC	TTGAGTTCGCTGTCCAACCA
Opa1	GCGGGTACACCTGGAGAAAC	GCAGAAGTTCTTCCTGAAGTTGG
Gapdh	ATGGCCTTGTAGACACCTTGG	ATGGCCTTGTAGACACCTTGG

Antibodies used in this study

Protein	Source	Identifier
Adiponectin	GeneTex	GTX112777
FABP4	abcam	ab92501
GAPDH	Millipore	MAB374
IFI27	GeneTex	GTX66233
Cleaved-caspase3	Cell Signaling Technologies	#9661
p62	MBL	PM045
LC3	Cell Signaling Technologies	#2775
Cyclin D1	Cell Signaling Technologies	#2922
PCNA	Cell Signaling Technologies	#2586
OXPHOS	abcam	ab110413
PPARγ	Santa Cruz	sc-7273
FAS	Cell Signaling Technologies	#2497
